# Sorbitol and *PKC1* overexpression alleviate temperature sensitivity in chaperonin mutants of *Saccharomyces cerevisiae*

**DOI:** 10.17912/micropub.biology.000440

**Published:** 2021-08-05

**Authors:** Aswathy Narayanan, M. Anaul Kabir

**Affiliations:** 1 School of Biotechnology, National Institute of Technology Calicut Kerala; 2 Molecular Mycology laboratory, Jawaharlal Nehru Centre for Advanced Scientific Research, Bangalore, Karnataka

## Abstract

CCT (Chaperonin containing TCP-1) is a constitutively expressed eukaryotic chaperonin complex involved in the proper folding of proteins like actin and tubulin. Temperature sensitive mutants of CCT complex have been employed in various genetic screens, acting as models to study human CCT, the defects of which are implicated in disease conditions like neurodegeneration. Mutants of CCT complex are sensitive to cell wall stress agents. In this study, we have tested the effects of sorbitol and protein kinase C overexpression on the temperature sensitivity of *cct* mutants. We report that both the factors alleviated temperature sensitivity of* cct* mutants, indicating the possible role of CCT in maintaining cell wall integrity in *S. cerevisiae*.

**Figure 1. Suppressors of temperature sensitivity in CCT complex chaperonin mutants:**
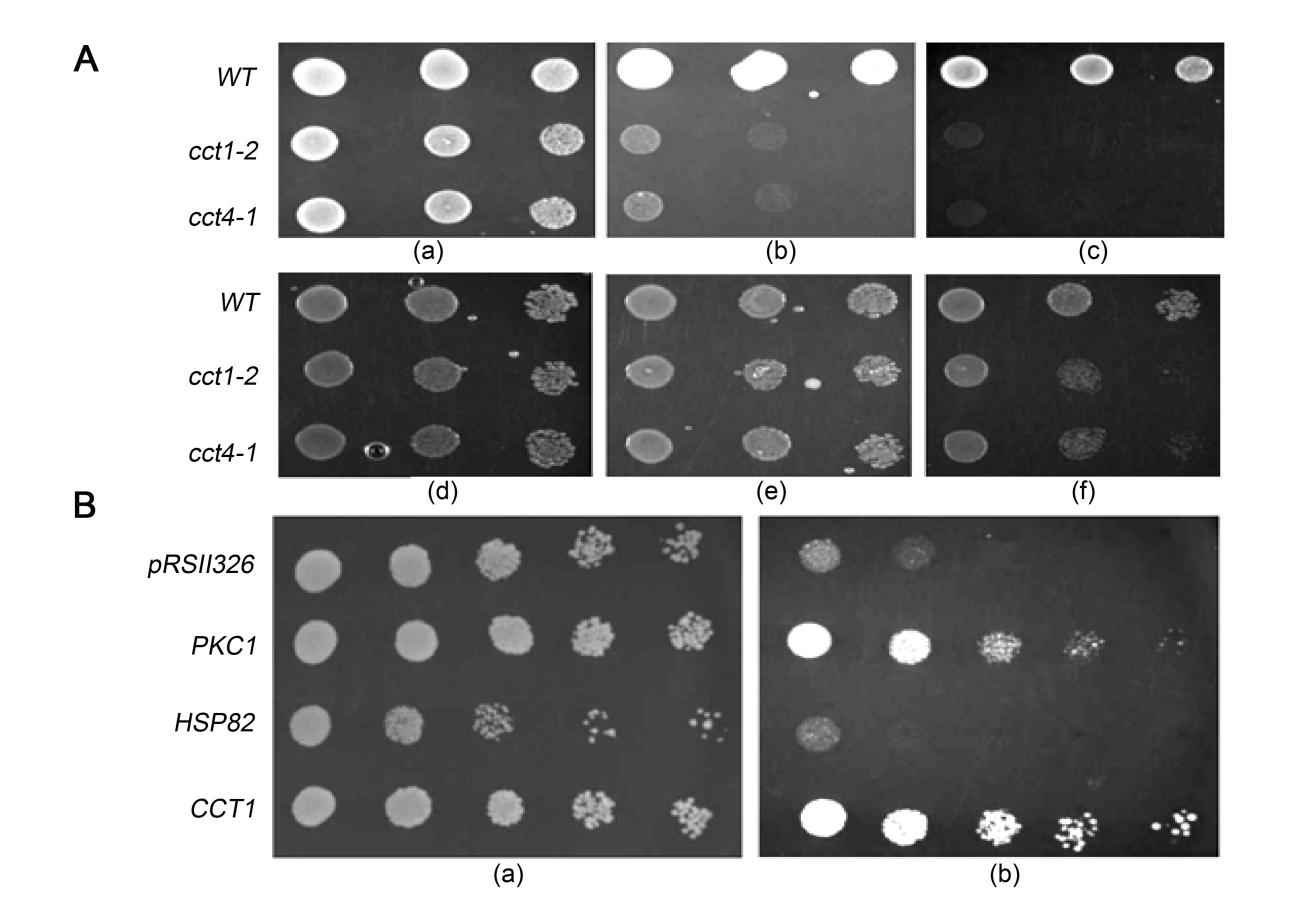
A) Effect of osmotic stabilizer on the temperature sensitivity of *cct* mutants. Strains B-8728 (WT), DUY326 (*cct1-2*) and DDY229 (*cct4-1*) spotted on YPD and incubated at (a) 30°C (b) 35°C and (c) 37°C. Strains B-8728 (WT), DUY326 (*cct1-2*) and DDY229 (*cct4-1*) spotted on YPD+1M Sorbitol and incubated at (d) 30°C (e) 35°C and (f) 37°C. B) Effect of *PKC1* and *HSP82* overexpression on temperature sensitivity of *cct1-2*. The mutant *cct1-2* transformed with pRSII326 (empty vector), *PKC1*, *HSP82*, and *CCT1* spotted on uracil-omission media after serial dilution and incubated at (a) 30°C and (b) 37°C.

## Description

TRiC (TCP-1 Ring Complex) or CCT (Chaperonin Containing TCP-1) is a multi-subunit, double-ringed, ATP-driven cytosolic eukaryotic chaperonin complex that is constitutively expressed and facilitates the folding of several proteins like actin and tubulin (Frydman *et al.* 1992; Llorca *et al.* 1999; Berger *et al.* 2018). Its role in many cellular processes like differentiation, viral replication, and heart contractility were elucidated recently (Melkani *et al.* 2017; Knowlton *et al.* 2018; Ohhara *et al.* 2019). Its functional defects are also associated with neurodegenerative diseases and cancer (Tam *et al.* 2006; Boudiaf-Benmammar *et al.* 2013). Temperature sensitive mutants of *Saccharomyces cerevisiae* CCT have been used in different studies, especially those involving genetic screens to identify CCT-interacting proteins (Kabir and Sherman 2008). The *CCT1*–*CCT8* genes encode eight different subunits of the complex; the mutant allele *cct1-2* has G423D replacement in the conserved ATP binding/hydrolysis domain in the subunit Cct1p. *cct4-1* (G345D), the mutation in subunit Cct4p, affects ATP binding, intra and inter-ring co-operativity (Shimon *et al.* 2008). *cct1-2* and *cct4-1* grew well at 30°C (permissive temperature), showed retarded growth at 35°C (semi-permissive temperature) and failed to grow at 37°C (restrictive temperature).

Their sensitivity to cell wall antagonists like SDS and congo red are suggestive of cell wall defects in *cct* mutants (Narayanan *et al.* 2016). Are the temperature sensitivity and cell wall defects linked in *cct* mutants? To answer this, we studied the effects of two different suppressors of cell wall defects on the temperature sensitivity of *cct* mutants.

Osmotic stabilisers like sorbitol are known to rescue cell-wall integrity mutants by preventing cell lysis (Ribas *et* *al.* 1991). We observed that the growth of *cct1-2* and *cct4-1* mutants was restored in presence of 1M sorbitol both at the semi-permissive and restrictive temperatures (Fig. 1A). Mutants in MAPK-activation pathway undergo cell lysis at elevated temperatures, due to the unrepaired cell wall defects (Kamada *et al.* 1995). The osmoremedial nature of temperature sensitivity in the *cct* mutants prompted us to study the role of cell wall biogenesis in temperature sensitivity. Towards this end, we overexpressed the *PKC1* gene in *cct1-2*. The *PKC1* gene in *S. cerevisiae* encodes protein kinase C, homolog of human protein kinase C, an important enzyme in the maintenance of cell wall integrity (Levin 2005). Overexpression of *PKC1* resulted in the rescue of the temperature-sensitive phenotype of *cct* mutants at 35°C and 37°C (Fig. 1B).

Wild type copy of the *CCT1* gene was also overexpressed that served as the positive control; the overexpression enabled growth at 35°C and 37°C, confirming that the defects observed in the mutant were indeed due to compromised CCT function. *HSP82*, a yeast *HSP90* homolog, was overexpressed in *cct1-2* to check if more copies of another chaperone can reverse the defects in the mutant. But, unlike *PKC1*, *HSP82* on overexpression failed to make an impact on the temperature sensitivity of *cct* mutants. Similar observations were made for *HSP40* and *HSP90*– overexpression of *PKC1* was found to ameliorate the temperature sensitivity in these heat shock protein mutants (Wright *et al.* 2007). Our results suggest the role of CCT, a constitutively expressed cytosolic chaperonin, in maintaining cell wall integrity in *S. cerevisiae*.

## Methods

Yeast extract (1%)- Peptone (2%)- Dextrose (2%) (YPD) medium was used to grow the yeast strains.The *cct1-2* and *cct4-1* strains were grown at different temperatures in media with and without sorbitol to study the effect of osmotic stabilizers. DUY326 was transformed with pFR22 (YEpU-*PKC1*) and pCMS154-*HSP82* for the overexpression of *PKC1* and *HSP82*, respectively (Wright *et al.* 2007) using the lithium acetate/PEG method. The transformants were selected on uracil-omission medium. The plates were incubated at 30 °C for two days and the transformants were subcultured in uracil-omission medium. Approximately equal number of cells of the transformants were serially diluted, spotted on YPD and incubated at different temperatures to study the phenotype.

## Reagents

**Table:** 

Plasmid	Description	Reference/Source
pRSII326	2- micron based multicopy plasmid with *URA3*	Chee *et al.* 2012
YEpU-PKC1	*PKC1* cloned in YEp352	Wright *et al.* 2007
pCMS154-HSP82	*HSP82* cloned in pRS426	Wright *et al.* 2007
pAB2477	*CCT1 URA3 CEN/ARS*

**Table:** 

Strain	Genotype	Figure	Source
B-8728	*MAT***a** *ura3-52 trp1-*Δ*63 leu2-*Δ*1 GAL2*	Figure 1A	Lin *et al.* 1997
DUY326	*MAT*a *ura3-52 lys2-801 ade2-201 cct1-1 leu2-3,-112*	Figure 1A	T C Huffaker
DDY229	*MAT***a** *cct4-1 leu2-3,112 lys2-801 ura3-52*	Figure 1A	D G Drubin
D094	DUY326 transformed with pAB2477	Figure 1B	This study
D352	DUY326 transformed with pRS11326	Figure 1B	This study
D353	DUY326 transformed with YEpU-PKC1	Figure 1B	This study
D354	DUY326 transformed with pCMS154-HSP82	Figure 1B	This study
